# Supplementary material for: Injectable Lipid Emulsion and Clinical Outcomes in Patients Exclusively Receiving Parenteral Nutrition in an ICU: A Retrospective Cohort Study Using a Japanese Medical Claims Database
Source: Nutrients. 2023 Jun 19;15(12):2797. doi: 10.3390/nu15122797 (PMC10304446; doi:10.3390/nu15122797)
Supplement: Supplementary file 1 [file nutrients-15-02797-s001.zip › Supplementary materials_revise2.pdf]

Supplementary Table S1 Classification of primary diagnoses

Diagnosis	International Classification of Diseases, 10th revision (ICD-10) Codes
Sepsis	A02.1, A20.7, A22.7, A26.7, A32.7, A40-A41, A42.7, B37.7
Neoplasms	C00-D48, excluding D10-D36
Diseases of the nervous system	G00-G99
Ischemic heart disease	I20-I25
Heart failure	I50
Cerebrovascular diseases	I60-I69
Other circulatory system diseases	I00-I15, I26-I49, I51-I52, I70-I99
Pneumonia	J10.0, J11.0, J12-J18, J85.1
Interstitial respiratory diseases	J80-J84
Other respiratory diseases	J00-J09, J10.1-J10.8, J11.1-J11.8, J20-J70, J85.0, J85.2-J85.3, J86-J99
Diseases of the digestive system	K00-K93
Kidney diseases	N00-N29
Injury, poisoning and certain other consequences of external causes	S00-S99, T00-T14, T20-T65
Other	Other than above

Supplementary Table S2 Classification of surgical procedures

Category of Surgery	Japan-specific Surgical Codes
Cardiovascular	K538-K6105, K613-K6173, K619-628, Excluding K601 and K602
Gastroenterological	K5201-K5372, K630-K663, K665-K7424
Cerebrovascular	K145-K181
Respiratory	K488-K5181
Orthopedic	K023-K144
Urological/Gynecological	K757-K8903
Multiple surgeries	More than one surgery in categories above
Other	Excluding above categories, K601, and K602
No surgery	Not applicable to any of codes above, or **no** record of surgery under general or lumber spinal anesthesia

Supplementary Table S3 Characteristics of patients by subgroups

Characteristics	Categories	Patient Subgroups		
		**ILE-only**	**ILE+ propofol**	**Propofol-only**
		**n = 1073**	**n = 541**	**n = 6847**
**Age**, *years*, n (%)	< 60	124 (11.6)	78 (14.4)	1511 (22.1)
	60–69	176 (16.4)	114 (21.1)	1473 (21.5)
	70–79	348 (32.4)	184 (34.0)	2144 (31.3)
	80–89	346 (32.2)	139 (25.7)	1545 (22.6)
	≥ 90	79 (7.4)	26 (4.8)	174 (2.5)
**Sex**, n (%)	Male	702 (65.4)	391 (72.3)	4585 (67.0)
	Female	371 (34.6)	150 (27.7)	2262 (33.0)
**Body mass index,** *kg/m* ^{2}, n (%)	< 16	122 (11.4)	40 (7.4)	277 (4.0)
	16–18.5	193 (18.0)	83 (15.3)	751 (11.0)
	18.5–22.5	389 (36.3)	201 (37.2)	2365 (34.5)
	22.5–25	193 (18.0)	110 (20.3)	1592 (23.3)
	≥ 25	176 (16.4)	107 (19.8)	1862 (27.2)
**Beds in admission hospital**, n (%)	< 200	37 (3.4)	21 (3.9)	304 (4.4)
	≥ 200, < 500	677 (63.1)	288 (53.2)	3776 (55.1)
	≥ 500	359 (33.5)	232 (42.9)	2767 (40.4)
**Admission year**, n (%)	2010–2011	36 (3.4)	19 (3.5)	244 (3.6)
	2012–2013	119 (11.1)	83 (15.3)	648 (9.5)
	2014–2015	214 (19.9)	104 (19.2)	1452 (21.2)
	2016–2017	277 (25.8)	151 (27.9)	2057 (30.0)

	2018–2019	363 (33.8)	163 (30.1)	2116 (30.9)
	2020	64 (6.0)	21 (3.9)	330 (4.8)
**Primary diagnosis**[1], n (%)	Sepsis	64 (6.0)	34 (6.3)	472 (6.9)
	Neoplasm	167 (15.6)	94 (17.4)	803 (11.7)
	Diseases of the nervous system	33 (3.1)	12 (2.2)	170 (2.5)
	Ischemic heart disease	29 (2.7)	20 (3.7)	644 (9.4)
	Heart failure	44 (4.1)	9 (1.7)	287 (4.2)
	Cerebrovascular disorders	63 (5.9)	32 (5.9)	652 (9.5)
	Other circulatory system diseases	97 (9.0)	78 (14.4)	1428 (20.9)
	Pneumonia	64 (6.0)	20 (3.7)	173 (2.5)
	Interstitial respiratory diseases	32 (3.0)	31 (5.7)	170 (2.5)
	Other respiratory diseases	99 (9.2)	20 (3.7)	264 (3.9)
	Diseases of the digestive system	249 (23.2)	115 (21.3)	960 (14.0)
	Kidney diseases	20 (1.9)	5 (0.9)	84 (1.2)
	Injury, poisoning, other consequences external causes	36 (3.4)	18 (3.3)	341 (5.0)
	Other	76 (7.1)	53 (9.8)	399 (5.8)
**Charlson Comorbidity Index**, n (%)	0	511 (47.6)	266 (49.2)	3521 (51.4)
	1-2	397 (37.0)	190 (35.1)	2375 (34.7)
	≥ 3	165 (15.4)	85 (15.7)	951 (13.9)
**Barthel Index,** n (%)	100	247 (23.0)	151 (27.9)	1733 (25.3)
	65–95	40 (3.7)	35 (6.5)	237 (3.5)

	45–60	45 (4.2)	15 (2.8)	208 (3.0)
	25–40	15 (1.4)	14 (2.6)	120 (1.8)
	5–20	65 (6.1)	36 (6.7)	241 (3.5)
	0	512 (47.7)	195 (36.0)	3335 (48.7)
	NA	149 (13.9)	95 (17.6)	973 (14.2)
**Japan Coma Scale**, n (%)	0	589 (54.9)	325 (60.1)	3766 (55.0)
	1–3	206 (19.2)	116 (21.4)	1096 (16.0)
	10–30	99 (9.2)	37 (6.8)	574 (8.4)
	100–300	179 (16.7)	63 (11.6)	1411 (20.6)
	NA	0 (0.0)	0 (0.0)	0 (0.0)
**Surgery**[2], n (%)	Cardiovascular	62 (5.8)	59 (10.9)	1092 (15.9)
	Gastroenterological	366 (34.1)	199 (36.8)	1544 (22.6)
	Cerebrovascular	40 (3.7)	21 (3.9)	376 (5.5)
	Respiratory	6 (0.6)	3 (0.6)	32 (0.5)
	Orthopedic	4 (0.4)	3 (0.6)	33 (0.5)
	Urological/Gynecological	7 (0.7)	3 (0.6)	31 (0.5)
	Multiple surgeries	6 (0.6)	5 (0.9)	51 (0.7)
	Other	28 (2.6)	19 (3.5)	147 (2.1)
	No surgery	554 (51.6)	229 (42.3)	3541 (51.7)
**Prescription/treatment**[3], n (%)	Catecholamines	744 (69.3)	430 (79.5)	5345 (78.1)
	Transfusion	562 (52.4)	331 (61.2)	4236 (61.9)
	Albumin	596 (55.5)	361 (66.7)	4075 (59.5)
	Renal replacement therapy	210 (19.6)	136 (25.1)	1879 (27.4)

	Intra-aortic balloon pump	35 (3.3)	26 (4.8)	859 (12.5)
	Plasmapheresis	1 (0.1)	4 (0.7)	39 (0.6)
	ECMO	12 (1.1)	19 (3.5)	500 (7.3)
	Nutritional support team	56 (5.2)	32 (5.9)	85 (1.2)
	Rehabilitation[4]	620 (57.8)	278 (51.4)	2940 (42.9)
**Parenteral nutrition**[5], median (Q1, Q3)	Energy, *kcal/kg*	14.1 (10.3, 19.1)	15.8 (11.7, 20.4)	9.8 (6.2, 14.5)
	Amino Acid, *g/kg*	0.41 (0.23, 0.59)	0.42 (0.25, 0.61)	0.19 (0.00, 0.38)
	Lipid, *g/kg*	0.19 (0.11, 0.29)	0.28 (0.19, 0.41)	0.12 (0.06, 0.20)
	Carbohydrate, *g/kg*	2.7 (1.8, 3.7)	2.9 (2.0, 3.7)	1.9 (1.1, 2.9)
**Days without nutrition from day 8**[6]**,** median (Q1, Q3)		0.0 (0.0, 0.0)	0.0 (0.0, 1.0)	0.0 (0.0, 0.0)

Abbreviations: ECMO, extracorporeal membrane oxygenation; Q1, first quartile; Q3, third quartile.
[1] Diagnoses identified using International Statistical Classification of Diseases and Related Health Problems, 10th revision.
[2] Between day of hospital admission and day of intensive care unit admission, based on Japan-specific codes.
[3] During days 1 to 7 of intensive care unit admission. Day 1 regarded as day of intensive care unit admission.
[4] Feeding therapy and/or rehabilitation for cardiac microvascular, cerebrovascular, disuse syndrome, locomotor, and/or respiratory diseases.
[5] Medians of mean prescribed daily doses during days 1 to 7 of intensive care unit admission.
[6] Days without nutrition (oral intake, enteral nutrition, or parenteral amino acids/lipid) from day 8 to day of discharge or in-hospital death.
